# Molecular Identification of Endophytic Fungi from Banana Leaves (*Musa* spp.)

**DOI:** 10.21315/tlsr2018.29.2.14

**Published:** 2018-07-06

**Authors:** Latiffah Zakaria, Wan Nuraini Wan Aziz

**Affiliations:** School of Biological Sciences, Universiti Sains Malaysia, 11800 USM Pulau Pinang, Malaysia

**Keywords:** Endophytic Fungi, Banana Leaves, *Musa* spp., ITS Sequences, Kulat Endofitik, Daun Pisang, *Musa* spp., Jujukan ITS

## Abstract

Endophytic fungi are part of microbial community found in various types of plant tissues including the leave, and display a range of symbiotic interactions with the plant host. In this study, endophytic fungi isolated from banana leaves were identified using ITS (Internal Transcribed Spacer region) sequences of which 10 genera comprising 17 species were molecularly identified. Endophytic fungal species identified were *Nigrospora oryzae*, *Nigrospora sphaerica*, *Colletotrichum gloeosporioides*, *Colletotrichum siamense*, *Fusarium equiseti*, *Fusarium chlamydosporum*, *Phoma sorghina*, *Pestalotiopsis oxyanthi, Pestalotiopsis theae, Pestalotiopsis eugeniae, Penicillium steckii, Penicillium purpurogenum, Bipolaris papendorfii, Bipolaris* sp., *Lasidiodiplodia theobromae, Cochliobolus intermedius* dan *Aspergillus niger.* The present study showed that several endophytic fungal genera/species are common plant pathogen and there is a possibility that these endophytes can become pathogenic. Some of the fungal endophyte might be mutualist or saprophyte.

## Abstract (ms)

Kulat endofitik merupakan sebahagian daripada komuniti mikrob dalam pelbagai jenis tisu tumbuhan termasuk daun, dan memaparkan pelbagai interaksi simbiotik dengan tumbuhan perumah. Dalam kajian ini, kulat endofitik dipencilkan daripada daun pisang, telah dikenal pasti menggunakan jujukan ITS (Kawasan Penjarak Dalaman) di mana 10 genus yang terdiri daripada 17 spesies telah dikenal pasti secara molekul. Spesies kulat endofitik yang dikenal pasti adalah *Nigrospora oryzae*, *Nigrospora sphaerica*, *Colletotrichum gloeosporioides*, *Colletotrichum siamense*, *Fusarium equiseti*, *Fusarium chlamydosporum*, *Phoma sorghina*, *Pestalotiopsis oxyanthi, Pestalotiopsis theae, Pestalotiopsis eugeniae, Penicillium steckii, Penicillium purpurogenum, Bipolaris papendorfii, Bipolaris* sp., *Lasidiodiplodia theobromae, Cochliobolus intermedius* dan *Aspergillus niger.* Kajian ini menunjukkan bahawa beberapa genus atau spesies kulat endofitik merupakan patogen tumbuhan yang lazim dan ada kemungkinan bahawa kulat endofitik ini boleh menjadi patogenik. Sebahagian kulat endofit ini mungkin mutualis atau saprofit.

Endophytic fungi reside asymptomatically in internal tissues of plants and form integral part of microbial community associated with various types of plants including crop plants, trees, herbs, shrubs, grasses, ferns as well as lichens and mosses (Zhang *et al.* 2006). Complex relationship or interaction exists between the endophyte and the plant host, which include commensalism, parasitism and mutualism (Sieber 2007). The interaction is often regarded as ‘plastic’ depending on the developmental stage and nutritional status, genetic dispositions between the endophytes and the host as well as environmental factors (Redman *et al*. 2001; Schulz & Boyle 2005). Thus, the interaction can change from mutualism to parasitism.

Based on a study by Brown *et al.* (1998) on pathogenic taxa of wild banana (*Musa acuminata*)*,* many potential pathogenic genera or species were encountered as endophytes. Some of the endophytes are latent pathogens of which the plant does not show disease symptoms when infected by the pathogen, but produce the disease symptoms when prompted by among others alteration of host physiology and changes in environmental and nutritional conditions (Verhoeff 1974). Similar observation might also occur in edible *Musa* spp. whereby endophytic fungi from the leaves are potential pathogenic genera or species.

For endophytic fungal studies, Internal Transcribed Spacer (ITS) region is frequently used for molecular identification as the region is recommended as universal DNA barcode marker for fungal identification (Schoch *et al*. 2012; Sun & Guo 2012). The use of ITS region as a marker has many advantages including availability of universal primers and databases, sufficient fragment length and high successful rate of amplification among all fungal lineages (Vilgalys 2003; Nilsson *et al*. 2009).

There is a possibility that some of the fungal endophyte resides in banana leaves are common genera or species of plant pathogens and some are saprophytes. Thus, the present study was conducted to isolate and molecularly identified endophytic fungi from leaves of *Musa* spp. to determine the endophytic and pathogenic fungal genera/species reside in banana leaves.

Symptomless banana leaves (*Musa* spp.) were obtained from banana trees at a banana farm in Balik Pulau, Kg Perlis, Pulau Pinang; banana trees from small garden near Bakti Permai hostel and banana trees near School of Biological Science plant house, Universiti Sains Malaysia main campus, Pulau Pinang. Young and healthy leaves were sampled and only one banana leave was chosen from the tree of which the estimated age of the banana plants were below six months. The samples were placed in plastic bags and brought to the laboratory to be processed. All the banana leaves were washed thoroughly under running tap water for 24 h and dried before isolation of endophytic fungi.

Isolation of endophytic fungi was carried out using surface sterilisation technique. After the banana leaves were thoroughly dried, the leaves were cut into 1 cm segment using a sterile scalpel. The pieces of banana leaves were sterilised by soaking in 2% sodium hypochlorite for 3 min, rinse in sterile distilled water for 1 min, blotted dried using sterilised filter paper to remove excess water.

After the banana leave pieces were thoroughly dried, imprint method was carried out by pressing the sterilised leaves segment gently onto the surface of Potato Dextrose Agar (PDA) to confirm the efficacy of the surface sterilisation technique and also to confirm only fungal endophyte were isolated. The absence of any fungal growth on the imprint plate showed that the surface sterilisation technique applied was effective in removing the surface fungi or epiphyte (Schulz *et al*. 1993).

The leaves segments were then transferred onto PDA and incubated at 25±1°C in a sterilised container. Four leaf segments were plated onto one PDA and 30 leaves were used for isolation. The PDA plated with the leaves segments were incubated for 1–4 days or until there was visible mycelium growth from the leave tissues. The mycelium grew from the banana leaves tissue were sub-cultured onto new PDA plates.

For DNA extraction, the fungal isolates were cultured on the surface of dialysis membrane on PDA, incubated for 5–7 days at 25±1°C or until there was visible mycelia growth. Invisorb® Spin Plant Mini Kit (STRATEC Molecular GmbH, Berlin, Germany) was used for DNA extraction according to the manufacturers’ instructions.

ITS regions were amplified using ITS1 (5′-TCC GTA GGT GAA CCT GCG G- 3′) and ITS4 (5′-TCC TCC GCT TAT TGA TAT GC- 3′) primers (White *et al*. 1990). PCR reaction mixture was prepared in 25 μl reaction containing 4 μL 1X PCR buffer, 4 μl 3.5 mM MgCl_2_, 0.5 μl of 0.16 mM of dNTP mix (Promega, Seattle, WA, USA), 0.15 μl of 1.75 unit of GoTaq® DNA polymerase (Promega), 4 μl of 0.0275 μM ITS 1 primer, 4 μl of 0.028 μM ITS 4 primer, 0.3 μl template DNA and 8.05 μl of ddH_2_O to make up a total volume of 25 μl. Paraffin oil (25 μl) was overlaid on each reaction. PCR was performed in MyCycler^™^ Thermal Cycler (Bio-RAD Hercules, CA, USA) with an initial denaturation at 95°C at 2 min followed by 35 cycles of 30 s denaturation at 95°C, 30 s annealing at 55°C and 1 min extension at 72°. Final extension for 10 min at 72°C was performed after the cycles ended.

After PCR, electrophoresis was run to detect the PCR product by using 1.0% agarose gel. Negative control which has no template DNA was used to detect any contamination. One μl of the PCR product was mixed with 3 μl 6X loading dye (ThermoFisher, Waltham, MA USA) and loaded in 1.0% agarose gel. The electrophoresis was run for 70 min at 80 V and 400 mA. PCR products were sent for sequencing to a service provider.

After sequencing, the sequences were aligned by using BioEdit Sequence Alignment Editor Version 7.0.5 software by Hall (1999) to obtain consensus sequences. The consensus sequences were then compared with other DNA sequences in GenBank using basic local alignment search tool (BLAST) in National Centre for Biotechnology Information (http://www.ncbi.nlm.nih.gov/). Identification of the isolates was based on the highest similarity of the BLAST search.

From 30 leaves segment, 28 endophytic fungal isolates were recovered. Based on BLAST search, the isolates were identified into 10 genera and 17 species with percentage of similarity from 97% to 100% (Table 1). Many of the fungal genera including *Pestalotiopsis, Colletotrichum, Nigrospora, Cochliobolus, Fusarium* and *Lasiodiplodia* are common endophytic fungi as well as plant pathogenic fungi. Among the fungal species identified, pathogens of *Musa* spp. were also identified such as *Colletotrichum gloeosporioides,* causal pathogen of anthracnose (Jones & Slabaugh 1998), *Lasidiodiplodia theobromae*, causal pathogen of crown rot (Jones & Slabaugh 1998) and *Nigrospora sphaerica*, causal pathogen of squirter disease of banana (Jones & Slabaugh 1998). In a study by Mohamed Abdalla *et al.* (2016), *Nigrospora* sp. and *Pestalotiopsis* sp. are saprophytes on organic banana fruits. The results of the present study suggested that endophytic stage may be important in the life cycles of some banana pathogens which conform to the statement by Brown *et al.* (1998).

Seven isolates of *Pestalotiopsis* spp. from banana leaf were identified as *Pes. thaea* (n = 3)*, Pes. eugeniae* (*n* = 2) and *Pes. oxyanthi* (*n* = 2). So far only *Pes. theae* is associated with banana of which the species was reported causing banana fruit rot (Ketabchi 2014). In nature, endophytic *Pestalotiopsis* is considered as a main part of *Pestalotiopsis* community (Kumar & Hyde 2004; Liu *et al.* 2006) and endophytic *Pestalatiopsis* from banana leaves give more information on the occurrence of this species in nature. Endophytic *Pes. theae* and *Pes. oxyanthi* have been reported as endophyte of Podocarpaceae, Theaceae and Taxaceae in southern China (Wei *et al*. 2007). They also suggested that *Pestalotiopsis* species could have endophytic and pathogenic stages in their life cycle.

According to Domsch and Gams (1993), *Nigrospora* is well-adapted as endophyte in plant tissues. In the present study, two *Nigrospora* spp. were identified as *N. oryzae* (*n* = 3) and *N. sphaerica* (*n* = 2). Endophytic *N. oryzae* is dominant species isolated from banana leaves in Hong Kong (Brown *et al*. 1998). Nevertheless, *N. oryzae* can also be saprophyte on banana leave (Holliday 1980; Surridge *et al*. 2003). *Nigrospora sphaerica* is closely related to *N. oryzae* and have been reported as pathogen of *Musa* spp. (Allen 1970; Wallbridge, 1981; Jones & Slabaugh 1998) as well as endophyte of Palmae (Rodrigues 1994). *Nigrospora sphaerica* was also among fungal isolates recovered from crown area of banana fruits (Wallbridge 1981). These studies suggested that *N. oryzae* and *N. sphaerica* can occur as endophyte and pathogen on different parts of banana plant.

Two species of *Colletotrichum* were identified in this study, *C. gloeosporioides* (*n* = 3) and *C. siamense* (*n* = 1). *Colletotrichum gloeosporioides* was among dominant endophytes isolated from banana in Hong Kong (Brown *et al*. 1998). *Colletotrichum gloeosporioides* is also causal pathogen of anthracnose and leaf spot of banana, however endophytic *C. gloeosporioides* from wild banana did not cause leaf spots on banana leaves *in-vitro* (Photita *et al*. 2004). *Colletotrichum siamense* is commonly a pathogen on wide host ranges (Phoulivong *et al*. 2012). The only report of *C. siamense* as an endophyte was from Refaei *et al.* (2011) of which *C. siamense* was among the endophytic fungi isolated from *Rafflesia cantley.*

*Penicillium* spp. are commonly reported in studies of endophytic fungal assemblages of various types of plants and have been recovered from different plant parts (Nicoletti *et al*. 2014). From banana leave segments, four isolates of *Penicillium* were isolated comprising three isolates of *P. steckii* and one isolate of *P. purpurogenum. Penicillium steckii* has not been reported as endophyte of banana but this species has been reported as among endophytic *Penicillium* spp. associated with coffee plant (Vega *et al*. 2006). Endophytic *P. purpurogenum* is common in plants and has been isolated from twigs of *Ginkgo biloba* (Qiu *et al*. 2010), different plant parts of *Acorus calamus* (Shukla & Mishra 2012), and green leaves of *Ziziphu*s spp. (El-Nagerabi *et al*. 2013). *Penicillium purpurogenum* is also plant pathogen causing fruit rot (Gubler & Converse 1994; Bhadwal & Sharma 2011) and root rot (Avasthi *et al.* 2015). In the present study, endophytic *P. steckii* might be mutualist in banana leaves, and there is a possibility that endophytic *P. purpurogenum* is latent pathogen of banana.

*Fusarium* is among fungal genera that have been reported as endophyte of many plants and several endophytic *Fusarium* spp. have been reported to be associated with banana plants (Marin *et al*. 1996; Photita *et al*. 2001; Athman 2006; Latiffah & Nur Hidayah 2011). In this study, two isolates of *Fusarium* identified as *F. equiseti* and *F. chlamydosporum* were recovered from banana leaves. Nevertheless, there is no report of endophytic *F. equiseti* and *F. chlamydosporum* associated with banana. Endophytic *F. equiseti* has been recovered from root of *Lygeum spartum*, a Gramineae (Maciá-Vicente *et al.* 2008), leaves of soybean plants (Russo *et al.* 2016) and from Poaceae (Szécsi *et al*. 2013). As for endophytic *F. chlamydosporum,* the endophyte was recovered from healthy roots of *Dendrobium crumenatum* (Orchidaceae) (Siddiquee *et al*. 2010), cocoa branches (Rubini *et al.* 2005), green leaves of *Ziziphus* sp. (El-Nagerabi *et al*. 2013) and stem of *Tylophora indica* (Chaturvedi *et al*. 2014). According to Leslie and Summerell (2006), *F. equiseti* and *F. chlamydosporum* are saprophyte or secondary coloniser of disease plant part. Therefore, both endophytic *F. equiseti* and *F. chlamydosporum* reside in banana leaves could later become saprophyte or secondary coloniser as the leaves aged.

Two endophytic *Bipolaris*, *Bipolaris* sp. and *B. papendorfii* as well as one species of *Cochliobolus* identified as *C. intermedius* were isolated from banana leaves. *Cochliobolus* is the sexual stage or teleomorph of *Bipolaris* (anamorph). Another anamorph of *Cochliobolus* is *Curvularia*. Both genera are worldwide pathogens of mostly grasses (Poaceae), however there are some species of *Cochliobolus* and *Bipolaris* reported as endophytes with different plant species (Manamgoda *et al.* 2011). Endophytic *Cochliobolus* was recovered from leaves of a medicinal plant, *Sapindus saponaria* L. (Garcia *et al.* 2012), and endophytic *Bipolaris* was the most frequent genus recovered from *Piper hispidum*, a medicinal shrub (Orlandelli *et al.* 2012). Both endophytic *Bipolaris* and *Cochliobolus* have not been reported from banana plants but *Curvularia* has been reported as endophyte of *Musa* spp. by Photita *et al.* (2004) and they regarded *Curvularia* as latent pathogen of *Musa* spp. There ia a possibility that *Bipolaris* and *Cochliobolus* might become pathogen to banana leaves.

Endophytic *P. sorghina, L. theobromae* and *A. niger* were recovered from banana leave segments. *Phoma sorghina* and *L. theobromae* are common plant pathogens and *A. niger* is well-known spoilage fungus, however, the three fungal species have also been found as endophyte in many types of plant. Endophytic *P. sorghina* has been reported in association with *Tithonia diversifolia* (Asteraceae) (Borges & Pupo 2006), rice plant (Fisher & Petrini 1992) and leaves of maize (Szilagyi-Zecchin *et al*. 2016). For endophytic *L. theobromae,* the species has been found to be associated with Araucariaceae (Huang & Wang 2011), as part of endophytic fungal community of cacao (*Theobroma cacao* L.) (Rubini *et al*. 2005) and among endophytes isolated from two types of orchids, *Bulbophyllum neilgherrense and Pholidota pallida* (Kotian *et al*. 2013). Endophytic *A. niger* has been isolated from leaves of *Platanus orientalis* (Robl *et al*. 2015), *Acacia arabica* (Tamanreet *et al.* 2016) and *Mangifera indica* (Nayak 2015). Similar with other endophytic fungi in the present study, *P. sorghina* and *L. theobromae* have the potential to become pathogens to banana leaves. As for *A. niger*, this species might become saprophyte when the leaves aged which is similar with *Fusarium*, *Bipolaris* and *Cochliobolus* recovered in this study.

The results of the present study suggested that several endophytic species are potential pathogens which in a latent phase. Similar observation was also reported by Photita *et al.* (2004) of which several endophytc fungi from wild banana leaves were able to cause leaf spot disease. Several factors that might contribute endophyte to become pathogenic including when the host plant is stressed (Andrews *et al*. 1985), change in host susceptibility due to poor nutrient supply and excessive humidity (Fisher & Petrini 1992). According to Bayman (2006), any factors that can weaken the host plant’s ability to limit growth of fungal endophyte could allow certain endophyte to become pathogenic.

As a conclusion, endophytic fungi isolated from banana leaves were identified into 10 genera comprising 17 species, namely *N. oryzae, N. sphaerica, C. gloeosporioides, C. siamense, F. equiseti, F. chlamydosporum, Phoma sorghina, Pes. oxyanthi, Pes. theae, Pes. eugeniae, P. steckii, P. purpurogenum, B. papendorfii, Bipolaris* sp., *L. theobromae, Cochliobolus intermedius* and *A. niger.* The present study showed that several endophytic fungal genera/species are common plant pathogens and there is a possibility they might become pathogen. Some of the fungal endophytes might be mutualist or saprophyte. The information on fungal endophyte of banana leaves also contributes to the knowledge on the biodiversity of endophytic fungi in Malaysia.

## Figures and Tables

**Table 1:** Endophytic fungi isolated from banana leaves identified using ITS sequences.

No.	Isolate	Species identity (% similarity)	Location
1	USM1-2	*Nigrospora oryzae* (99)	Kg Perlis, Balik Pulau
2	USM1-3B	*N. oryzae* (99)	Kg Perlis, Balik Pulau
3	USM2-1A	*N. sphaerica* (100)	Kg Perlis, Balik Pulau
4	USM 2-1B	*N. sphaerica* (100)	Kg Perlis, Balik Pulau
5	USM 2-8A	*N. sphaerica* (100)	Kg Perlis, Balik Pulau
6	USM 2-6A	*Colletotrichum gloeosporioides* (99)	Kg Perlis, Balik Pulau
7	USM 5-4	*C. gloeosporioides* (99)	Bakti Permai, USM
8	USM 7-5	*C. gloeosporioides* (100)	Plant House, USM
9	USM 6-2	*C. siamense* (100)	Bakti Permai, USM
10	USM 3-2A	*Fusarium equiseti* (100)	Kg Perlis, Balik Pulau
11	USM 3-3	*F. chlamydosporum* (99)	Kg Perlis, Balik Pulau
12	USM 3-8A	*Phoma sorghina* (100)	Kg Perlis, Balik Pulau
13	USM 4-3	*Pestalotiopsis oxyanthi* (97)	Bakti Permai, USM
14	USM 5-7	*Pes. oxyanthi* (99)	Bakti Permai, USM
15	USM 5-2	*Pes. theae* (99)	Bakti Permai, USM
16	USM 5-3	*Pes. theae* (99)	Bakti Permai, USM
17	USM 5-5	*Pes. theae* (99)	Bakti Permai, USM
18	USM 5-3A	*Pes. eugeniae* (99)	Bakti Permai, USM
19	USM 7-2	*Pes. eugeniae* (99)	Plant House, USM
20	USM 8-10	*Penicillium steckii* (99)	Plant House, USM
21	USM 8-11	*P. steckii* (99)	Plant House, USM
22	USM 8-12	*P. steckii* (99)	Plant House, USM
23	USM 7-4	*P. purpurogenum* (100)	Plant House, USM
24	USM 9-7	*Bipolaris papendorfii* (100)	Plant House, USM
25	USM 8-1	*Bipolaris* sp. (100)	Plant House, USM
26	USM 1-3A	*Lasiodiplodia theobromae* (100)	Kg Perlis, Balik Pulau
27	USM 8-7	*Cochliobolus intermedius* (99)	Plant House, USM
28	USM 7-1	*Aspergillus niger* (100)	Plant House, USM
